# A new tool for studying waterfowl immune and metabolic responses: Molecular level analysis using kinome profiling

**DOI:** 10.1002/ece3.4370

**Published:** 2018-07-30

**Authors:** Giovanni Pagano, Casey Johnson, D. Caldwell Hahn, Ryan J. Arsenault

**Affiliations:** ^1^ Center for Bioinformatics and Computational Biology University of Delaware Newark Delaware; ^2^ Department of Animal and Food Sciences University of Delaware Newark Delaware; ^3^ USGS Patuxent Wildlife Research Center Laurel Maryland

**Keywords:** American black duck, Anatidae, cellular signaling pathways, immunometabolism, kinome, mallard duck, peptide array

## Abstract

Here, we describe the design of an *Anas‐*specific kinome peptide array that can be used to study the immunometabolic responses of mallard and American black duck to pathogens, contaminants, and environmental stress. The peptide arrays contain 2,642 unique phosphorylate‐able peptide sequences representing 1,900 proteins. These proteins cover a wide array of metabolic and immunological processes, and 758 Gene Ontology Biological processes are statistically significantly represented on the duck peptide array of those 164 contain the term “metabolic” and 25 “immune.” In addition, we conducted a comparison of mallard to American black duck at a genetic and proteomic level. Our results show a significant genomic and proteomic overlap between these two duck species, so that we have designed a cross‐reactive peptide array capable of studying both species. This is the first reported development of a wildlife species‐specific kinome peptide array.

## 1 INTRODUCTION

Many factors have been considered in assessing the precipitous decline of the iconic American black duck (*Anas rubripes*) in the mid‐Atlantic during the latter half of the 20th century (Grandy, 1983; Maryland_DNR, 2015). Once the most abundant duck species in the Chesapeake and Delaware Bays and a game species prized by hunters (Feierabend, 1984), the black duck's decline was suspected to be due to one or more changing environmental factors, including: (a) increased pollutants and contaminants (Gosnell et al., 2016; Ng, Szabo, Reilly, Barringer, & Smalling, 2016; Potter, Watts, La Guardia, Harvey, & Hale, 2009; Rattner, Fleming, & Bunck, 1989; Yonkos, Friedel, Perez‐Reyes, Ghosal, & Arthur, 2014); (b) increased land development entailing loss of wetland habitat and increased disturbance associated with human activity (Blazer et al., 2013; Coxon, Odhiambo, & Giancarlo, 2016); and (c) loss of traditional food items (Maryland_DNR, 2015).

Simultaneous with the decline of the black duck, the closely related mallard duck (*Anas platyrhyncho*s) increased significantly in abundance (Ankney, Dennis, & Bailey, 1987; Johnson & Sorenson, 1999). Wildlife biologists and ornithologists speculated that the two species differed physiologically in tolerance to changing environmental conditions (Heusmann, 1974). It is also possible that species hybridization between the American black duck and mallard have reduced the population of the distinct black duck population (Mank, Carlson, & Brittingham, 2004). Physiological mechanisms are likely to affect the ducks’ tolerance to these environment changes (Coluccy et al., 2015; Gasaway & Buss, 1972; Hayes & Wobeser, 1983), but no tool was available for a systematic assessment of the two duck species’ immune and metabolic responses.

Here, we report the first design of a kinome tool for use on wild species. The mallard genome was sequenced in 2013 (Huang et al., 2013), and we have constructed a species‐specific peptide array based on this information as well as all available proteomic data. The mallard genome was sequenced, because it was an economically important waterfowl species, and also because there is particular medical interest in the mallard. The mallard duck is an abundant natural host of the influenza A virus, which is a dangerous threat to public health (Lycett, Bodewes, & Pohlmann, 2016). Mallards harbor most subtypes of avian influenza currently known (Huang et al., 2013; Papp et al., 2017). The sequenced mallard genome provided the basis for designing and creating a species‐specific peptide array to examine immune and metabolic responses. This immunometabolic array allows us to examine the mallard immune and metabolic responses in much greater detail than has been possible previously.

We also report a mallard—black duck comparison, in a similar fashion to our work comparing turkeys and chickens (Arsenault, Trost, & Kogut, 2014). We have shown previously that peptide arrays have cross‐reactivity for congeneric species, even at the family level (Booth et al., 2010). We have thus constructed a peptide array based on the mallard genome and proteome that we show can be used for black duck based on the available published genetic information. We plan to compare immune and metabolic responses between the two *Anas* species, and to assess how the physiology of the two duck species differs with an eye to understanding their contrasting population trends in the mid‐Atlantic.

Studying different levels of the cell response can give us a better picture of how the black duck and mallard adapt to stresses like disease, and the cellular signaling mechanisms underlying these responses. One of the more popular methods for identifying biological responses by active cellular signal transduction pathways involves looking at levels of RNA expression in cells or tissue. However, the levels of RNA do not always correspond to a phenotypic change in cells, and some RNAs may not even be translated, due to mechanisms such as RNA silencing (Moazed, 2009). Transcriptome data analysis makes a number of assumptions in the context of pathway analysis and cell function. These assumptions include that the mRNA is stable, the mRNA is not silenced, the mRNA is translated into protein, the protein is properly folded, the protein is stable and not degraded before function, and finally that the protein is activated. It is more phenotypically relevant to look at changes directly at the protein level, and at mechanisms like post‐translational modifications (PTMs) of proteins. One class of PTMs is the addition and removal of phosphate groups to influence the function of proteins. Phosphorylation and dephosphorylation are ubiquitous in cell signaling and protein regulation, and the total activity of the cell's kinases (phosphorylation enzymes) is termed the kinome. Investigating the kinome could allow insights into biological response closer to phenotypic changes than the transcriptome (Chen, Stookey, Arsenault, & Napper, 2016).

**Figure 1:**
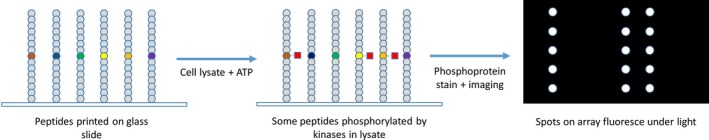
General protocol for using a species‐specific peptide array. Strings of amino acids containing kinase target sites (colored dots) are exposed to kinases, which recognize and phosphorylate the sites (red squares). After staining and imaging, phosphorylated sites appear as fluorescent dots, which can then be quantified and compared to a control or other treatments to find significant differences and active biological pathways

To study the phosphorylation/dephosphorylation of kinase targets in a species of interest, species‐specific peptide arrays have been developed for use in various organisms (Jalal et al., 2009). These arrays contain short (15 amino acid) peptides that represent the target site for specific kinases (see Daigle et al., 2014 for an example array). These sites have been shown to be acceptable substrates for kinases, with similar enzyme kinetics to the full protein (Zhu et al., 2000). Peptide arrays can be customized to different species, different signaling pathways, and are usable with a variety of treatment‐control or treatment–treatment combinations (Figure 1).

A previous study used the chicken and turkey proteomes along with the genome/proteome comparison software DAPPLE to determine the homology between these two species for the purpose of peptide array design (Arsenault et al., 2014). A comparison of the two proteomes revealed homology between orthologous proteins (83%) and between predicted phosphorylation sites (70%–75%). It was determined that this level of homology was not sufficient to confidently design a cross‐species chicken/turkey peptide array and an array was designed for each species individually. Here, we consider the similarity at a genetic and proteomic level between the black duck and mallard to determine whether there is enough homology to extrapolate mallard phosphorylation sites for a cross‐reactive array. Designing an array for both mallard and black duck presents an additional challenge, as the black duck data are limited to short protein sequences and genome sequences from barcode studies of different isolates. However, the complete mallard genome and proteome are available, and the mallard and black duck are more closely related to each other (c.f. Mank et al., 2004) than chicken and turkey.

This molecular tool can be used to examine physiological differences between mallard and black ducks that may explain the dramatic difference in their population trends. The tool creates a kinome profile (Shakiba et al., 2009) by utilizing a species‐specific peptide array, which measures phosphorylation changes at the protein level, thus characterizing cellular signaling events (Daigle et al., 2014). The peptide array has been in use in biomedical research for nearly two decades (Cahill, 2001; Cretich, Damin, Pirri, & Chiari, 2006; Davies et al., 2010) where it was applied to humans and to the mouse, a principal model species, but has only recently been available for animal research per se. The tool was applied first to agricultural animals, where it has been used to gain insight into such mechanisms as immune responses to specific infections in chickens (Kogut & Aresenault, 2017) and honeybees (Robertson et al., 2014) as well as metabolic response of cattle to stress during handling (Chen et al., 2016).

An important advance in immunological studies, and one of the first uses of this technology in avian species, resulted from the work on chicken infected with *Salmonella* demonstrating that the immune and metabolic systems are tightly interlocked, to the extent that it was concluded that the two should not be considered independent systems (Arsenault & Kogut, 2015). The peptide array used in these studies had incorporated both metabolic and immune signaling pathways, and this finding confirmed the utility of the array design in documenting major physiological responses to pathogens and other sources of stress.

Our approach to designing a species‐specific peptide array for kinome analysis has been carried out on numerous species to investigate a number of biological questions. In an avian species example, the peptide array data provided a new focus for research on eliminating chronic infection of chicken with *Salmonella,* a major public health and poultry industry problem. Investigators had been stymied because infected chickens are often asymptomatic, so they are sent to market. The mechanism underlying the chicken's lack of symptoms was identified (Kogut & Aresenault, 2017) using the first avian peptide array, a species‐specific immunometabolic array designed for the chicken. Examining chickens over the course of experimental infection with *Salmonella*, it was shown that the chicken initially mounts a strong inflammatory response following *Salmonella* infection, but following this classic immune defense, that is, a *resistance* type defense, the inflammatory response is downregulated within a few days, ceasing to attack the pathogen, and generating a *tolerating* response in the host. These data provide the insight immunologists need to recognize that the chicken's immune response changes predictably to a *tolerance‐type* defense and that allows the disease organism to persist at a low concentration. Thus, these peptide array data constitute a major insight to guide research by poultry immunologists in a different direction to address the chicken's use of this type of immune defense. Tolerance‐type immune defenses are well understood in plants and the agricultural literature, but have not been studied in animals until recently (Atkinson, Saili, Utzurrum, & Jarvi, 2013; Raberg, Sim, & Read, 2007).

Wildlife researchers are often concerned with environmental stressors, and this technique has been used previously to study stress responses in domestic animals. As stress causes negative effects on animal health and productivity, it is a priority for the livestock industry to reduce stress in cattle in all phases of handling and management. There is a need for cost‐effective ways to measure the stress response in individual cattle both as a guide to modifying handling practices and also to identify cattle with high‐stress tolerance to use in breeding programs. The traditional method to detect and measure stress is to measure serum cortisol levels, but these studies are expensive, require access to technical laboratory equipment, and yield inconsistent results. Using kinome analysis (Chen et al., 2016), the cattle stress response was analyzed at the molecular level, which resulted in a breakthrough by identifying an easier, less expensive method of assessing stress via measurement of glucose levels. The peptide array revealed that glucose metabolism was highly correlated with serum cortisol, thus is a good alternate indicator of stress, because glucose measurement is easier and less expensive.

Finally, the kinomic approach has also been applied to the consideration of population decline due to multifactorial environmental conditions, specifically the cause of population declines of North American honeybees (*Apis mellifera*) due to colony collapse disorder (Cox‐Foster et al., 2007; Paxton, 2010). This disorder, in which the *Varroa* mite infects bees and destroys the colony, arose relatively recently but has been very costly and has raised concerns that the disorder will spread throughout the continent and cripple the bee pollinator industry. Using a honeybee‐specific peptide array, Robertson et al. (2014) discovered that the mite infections are not the *cause* of the disorder, but instead are a *result* of a genetic predisposition to mite infection in some bees. This discovery guides research away from treating the infection and focuses research on identifying mite‐resistant bees and developing breeding programs with them. Peptide array data may yield a similar insight into a genetic predisposition in black ducks that limit their tolerance to changing environmental factors in the bays.

**Figure 2:**
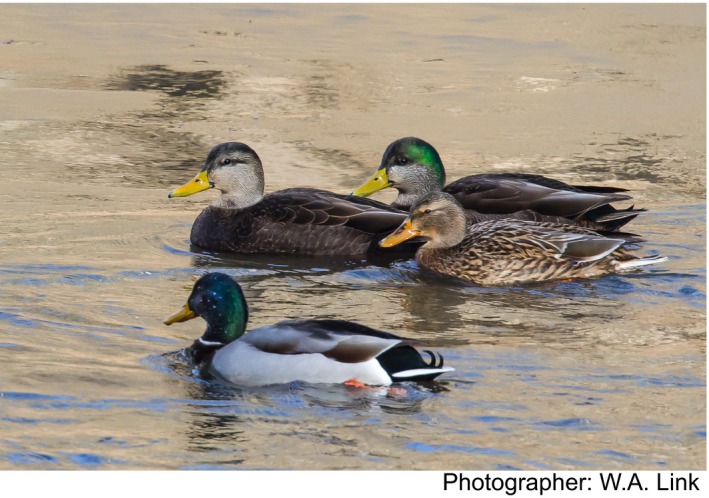
Mallard ducks, American black duck, and a hybrid duck. A male mallard duck is in foreground, a male American black duck is leading the group of three, and a female mallard is behind the American black duck, to its left. The hybrid (mallard × American black duck) is behind the American black duck, to its right and has a partially green head and a slight tail curl. Photographed by William A. Link on January 20, 2018 on the Patapsco River north of Baltimore Maryland, USA (latitude 39.309245, longitude −76.793080)

Now constructed, the *Anas*‐specific peptide arrays remain available at the University of Delaware Kinome Center and can be used for a study of immune and metabolic responses of mallard and black ducks to pathogens and to many environmental factors associated with degradation of their habitat in the Chesapeake and Delaware Bays which are likely to reveal why black duck is less tolerant than mallard to some or all of these factors (Figure 2).

## 2 MATERIALS AND METHODS

### 2.1 Comparison of black duck/mallard genome and proteome

Available proteomic data for the black duck were obtained from UniProt (http://www.uniprot.org/) (UniProt Consortium, 2016), and available genomic data were obtained from by searching All Databases of NCBI (https://www.ncbi.nlm.nih.gov/) for American black duck data available as of December 2016. In several cases, multiple entries for a gene from different isolates of black duck were present in NCBI, so a single isolate was chosen in each case. In total, sequences representing 29 different genes and 11 different proteins were found for black duck. The sequences were used in a BLAST (2.5.0) search (https://blast.ncbi.nlm.nih.gov/Blast.cgi; blastn for the genes, blastp for proteins) against mallard (BGI_duck_1.0), from NCBI, and the top hit for each sequence was chosen for comparison.

### 2.2 Prediction of mallard phosphorylation sites

The complete mallard proteome was downloaded from NCBI and was uploaded to the DAPPLE 2 server (http://saphire.usask.ca/saphire/dapple/index.html). The PhosphoSitePlus database (https://www.phosphosite.org; Hornbeck et al., 2015) was used as the set of known phosphorylation data, and only the top result for each predicted site was returned. The list of putative sites was narrowed down as described in the results section.

### 2.3 Conservation of putative mallard sites

In total, 2,642 peptides were chosen from the DAPPLE output for further analysis on the arrays. To identify sites with homologs in black duck, this list of sites was searched against the known black duck proteome using blastp. Two searches were conducted; one using the default BLAST parameters and one using default parameters optimized for short query sequences.

### 2.4 Final array design analysis

The STRING 10.5 protein–protein interaction database (Szklarczyk et al., 2017) was used to analyze the final mallard kinome peptide list by inputting the entire 2,642 peptide list as UniProt IDs. The STRING outputs generated are described in the results section below. This peptide list was sent to JPT Peptide Technologies (Berlin, Germany) for peptide synthesis and printing. Peptide array production was done on a contract basis with JPT Peptide Technologies (Berlin, Germany). Their production processes involves the synthesis of amino‐oxy‐acetylated peptides on cellulose membranes in a parallel manner using SPOT synthesis technology. Two droplets of 0.5 nl peptide solution were deposited per spot on aldehyde functionalized glass slides using the noncontact printer Nanoplotter GESIM. To determine intra‐assay technical variability in substrate phosphorylation, each peptide was printed with nine replicates. The physical dimensions of the array were 75 × 25 mm.

## 3 RESULTS

### 3.1 Homology of black duck and mallard

In total, 30 different genes and 11 different protein sequences were found in databases for the American black duck. The genes represent both intron and coding DNA sequence, and the total set contains both mitochondrial and genomic sequences. All the black duck sequences had very high homology to sequences from the mallard proteome or genome (above 97%). Table 1 summarizes the homology of the known genome and proteome. The results for the similarity search for each of the equivalent 11 proteins in mallard are shown in Table 2; each has a very high degree of similarity.

**Table 1:** Summary of homology between mallard and black duck

	# of sequences	% Identity	# of gaps
Genes	29	99.39	0
Proteins	11	99.86	2

*Note*

% identity represents the total amount of homology across all genes or proteins compared. # of gaps represents the number of gaps in the sequence where there is a missing nucleotide/amino acid, thus not caused by mismatched residues or bases.

**Table 2:** Black duck proteins homology to mallard

UniProt ID	Name	Length	Bit score	E‐value	%
Q68NC3_ANARU	Cytochrome c oxidase subunit 1 (Fragment)	230	448 bits (1,152)	3.00E‐162	99
Q7J4X4_ANARU	NADH‐ubiquinone oxidoreductase chain 2	346	667 bits (1,722)	0	100
O63740_ANARU	Cytochrome b (Fragment)	348	695 bits (1,794)	0	100
T2B123_ANARU	Melanocyte‐stimulating hormone receptor (Fragment)	261	525 bits (1,353)	0	100
T2B0T7_ANARU	Alpha‐enolase (Fragment)	39	88.6 bits (218)	6.00E‐23	100
B5L8W6_ANARU	Ornithine decarboxylase (Fragment)	29	60.1 bits (144)	6.00E‐15	100
T2B2J8_ANARU	Chromo‐helicase‐DNA binding protein (Fragment)	18	37.7 bits (86)	2.00E‐06	100
T2B1P5_ANARU	Nucleolin (Fragment)	11	35.4 bits (76)	2.00E‐05	100
T2AYZ2_ANARU	Alpha‐B crystallin (Fragment)	15	34.3 bits (77)	6.00E‐05	100
T2B0Z4_ANARU	Annexin A11 (Fragment)	8	27.8 bits (58)	0.01	100
T2B0F7_ANARU	N‐methyl D‐aspartate 1 glutamate receptor (Fragment)	8	31.6 bits (67)	3.00E‐04	100

One point to note with a few of the alignments is that exon–intron labeling appears to vary between black duck and mallard; what was identified as an intron region in black duck may be identified as an exon in mallard or vice versa. However, these cases were in close proximity on the gene (e.g., exon 4 versus intron 3), so any differences in naming may be due to differences in the annotation process.

### 3.2 Rationale for peptide selection

We utilized the program DAPPLE (Trost, Arsenault, Griebel, Napper, & Kusalik, 2013) to generate results representing mallard peptide sequence orthologs found from reference organisms (query species) which have well annotated genomes. Peptides refer to short amino acid sequences, 15 monomeric units (mer) in length. This set of orthologous peptides was used as a starting point in the establishment of a mallard immunometabolic kinome peptide array. A number of columns of data are provided as the DAPPLE output conveying what is known about the peptide sequences and phosphorylation sites (Table 3). The information provided in this table as well as outside sources (described below) was referenced and used in the development of a mallard immunometabolic kinome peptide array. The following procedures were used to truncate the list of peptides from the original DAPPLE output spreadsheet.

**Table 3:** DAPPLE output column descriptions

Column headings	Column description
Query^a^ accession	UniProt ID
Query description	Protein name
Query organism	Species
Query sequence	15‐mer sequence
Query site	Phosphorylated amino acid in sequence
Hit^b^ site	Phosphorylated amino acid in sequence
Hit accession	UniProt ID
Hit description	Protein name
Hit sequence	15‐mer sequence
Sequence differences	# of differences in 15‐mers between Query and Hit
Non‐conservative sequence differences	Only those 15mer sequence differences determined to be non‐conservative by BLOSUM62 matrix (≤0)
9‐mer sequence differences	# of differences in the center 9‐mer sequences
9‐mer non‐conservative sequence differences	Only those 9mer sequence differences determined to be non‐conservative by BLOSUM62 matrix (≤0)
Hit protein rank	Order of Hit protein following blastp
Hit protein E‐value	E‐value of match between Query and Hit protein
RBH?	Reciprocal Blast Hit. If reverse blast Hit‐to‐Query generates the same protein column will display “yes”
Low‐throughput references	# of literature citations using low‐throughput experimental techniques to confirm 15mer phosphorylation (e.g. western blot)
High‐throughput references	# of literature citations using high‐throughput experimental techniques to confirm 15mer phosphorylation (e.g. mass spectrometry)
Query GO terms	Gene ontology terms that describe query protein
Hit GO terms	Gene ontology terms that describe Hit protein
Query keywords	Keywords that describe query protein
Hit keywords	Keywords that describe query protein
Query reactome pathways	Reactome pathways containing query protein
Hit reactome pathways	Reactome pathways containing Hit protein

*Notes*

Column headings are DAPPLE outputs. Column description explains the results within these columns.

^a^Query refers to a species with a well annotated phosphoproteome. The data from this species are used to query the databases of the species of interest. ^b^Hit refers to the species of interest, in this case mallard.

1. Phosphorylation in eukaryotic cells occurs on the amino acids serine, threonine, and tyrosine. If the Hit sites, the orthologous kinase target site in the mallard peptide sequence showed any amino acid other than a serine, threonine, or tyrosine, these were eliminated from the DAPPLE output. This site would not be considered a phosphorylate‐able site, as it does not contain the correct amino acid residue.
2. Sequences lacking homology between species are less likely to have the same function in both species. The greater the sequence divergence, the more likely the site is to have a different function. Peptides showing five or more sequence differences between the 15‐mer query sequence and hit sequence were eliminated from the array design. Similarly, if three or more differences were observed between the center 9‐mer query sequence and the center 9‐mer hit sequence, the peptide was eliminated.
3. Kinase target sites with identical sequences can exist in multiple proteins. This may be due to different isoforms of a protein or proteins sharing specific phosphorylate‐able domains. In this case, one sequence would represent multiple proteins. Thus, any redundant 15‐mer sequences were removed from the array list. There is no risk of losing biological meaning by eliminating redundant sequences because when analyzing the data generated from the arrays one must look into site function and consider the existence of the same sequence in multiple proteins as well as all possible site functions.
4. An orthologous protein is identified by blasting the query protein against the hit species proteome, in this case mallard. We then did the reverse, blasting the mallard protein against the query proteome, in this case generally human. If the same protein is identified as the top hit in both blast results this is considered a reciprocal blast hit. Peptides without a reciprocal blast hit were removed from the array design.
5. The DAPPLE output contains Gene Ontology (GO) Terms (Ashburner et al., 2000 and Gene Ontology Consortium, 2016) annotated to the protein of interest. These describe the function of the protein. These GO Terms were searched for the terms “Immune” and “Metabolic” to identify peptides relevant to immunometabolic biological processes. Proteins not involved in these processes were eliminated from the array design.
6. Query peptides from human were preferred over those from rat and bovine. This was done to allow consistent analysis of the Hit results and because of the more detailed annotation of the human proteome. Other query species results were retained if they identified a known protein in the mallard as indicated by the presence of literature‐based annotations and characterization.
7. Various functionally similar proteins were present in the DAPPLE generated peptide list. The best annotated of these, namely those with the highest number of low‐throughput and/or high‐throughput references, were retained. Low‐throughput references refer to the number of literature citations using low‐throughput experimental techniques to confirm 15mer phosphorylation (e.g., western blot), high‐throughput indicates a technique such as mass spectrometry. Low‐throughput references were given priority over high‐throughput references.
8. Lastly, site relevance to a change in protein function was considered. Phosphosite and/or UniProt were referenced to determine site function. Phosphosite and UniProt were used to lookup the function of the phosphorylation site of the peptide. If the function was inconsequential for immune or metabolic signaling, the peptide was removed from the list.

### 3.3 Conservation of mallard sites in black duck

A blastp search with our final list of peptides was used to find homologous peptides in the known black duck proteins. Under default BLAST parameters, a total of 767 hits representing 322 peptides were found. When the BLAST was optimized for short sequences, a total of 1,946 hits representing 689 peptides were found. In both cases, the E‐values of the hits were mostly over 0.01, except for a pair of hits which have been noted and identified in Table 4. Many of these hits represent short matches against unrelated proteins, especially in the case of the BLAST optimized to short sequences. The strongest match in both searches was an exact match serine 272 of the metabolic enzyme alpha‐enolase (UniProt ID: U3IR52). Another strong match occurred with tyrosine 20 of the cytochrome c oxidase subunit 1 (UniProt ID: P50656). Other matches in the short search were between uncharacterized proteins in the mallard to short pieces of cytochromes and a melanocortin 1 receptor. Based on the partial annotations for the proteins provided on the UniProt web site, it seems unlikely that these uncharacterized proteins represent true homologs to the black duck. The inability to find these kinase recognition peptide sequences in the black duck is certainly more an issue of genetic and proteomic annotation than a lack of homology between these two species. As we have described when genetic and proteomic sequences are known in the black duck, they are approximately 99% similar to mallard.

**Table 4:** Mallard peptides with significant homology to black duck

Uniprot ID	Residue	Protein name	E‐value (regular)	E‐value (short)
U3IR52	S272	Alpha‐enolase	3.92E‐08	3.93E‐07
P50656	Y20	Cytochrome c oxidase subunit 1	7.85E‐04	0.002

*Note*

E‐value (regular) represents the E‐value under regular BLAST parameters. E‐value (short) represents the E‐value under BLAST parameters optimized to short sequences.

### 3.4 Biology of final array design

The final peptide array design contains 2,642 unique phosphorylate‐able peptide sequences representing 1,900 proteins (see Supporting Information Data S1). These proteins cover a wide array of metabolic and immunological processes. 758 GO Biological processes are statistically significantly represented on the duck peptide array (*p* < 0.05 FDR corrected); of those 164 contain the term “metabolic” and 25 “immune” (many more are related to individual immune responses). A selection of the top GO Terms from the peptide list can be seen in Table 5.

**Table 5:** Selection of GO term biological processes represented on immunometabolic duck array

GO ID^a^	Pathway description^b^	No. of proteins^c^	False discovery rate^d^
GO.0044281	Small molecule metabolic process	195	5.99E‐96
GO.0044710	Single‐organism metabolic process	223	3.18E‐68
GO.0044711	Single‐organism biosynthetic process	125	2.13E‐57
GO.0043436	Oxoacid metabolic process	104	1.64E‐53
GO.1901564	Organonitrogen compound metabolic process	129	1.65E‐51
GO.0044238	Primary metabolic process	271	4.55E‐43
GO.0006629	Lipid metabolic process	99	4.73E‐43
GO.1901575	Organic substance catabolic process	115	1.78E‐42
GO.1901566	Organonitrogen compound biosynthetic process	93	6.45E‐42
GO.0044255	Cellular lipid metabolic process	84	2.39E‐39
GO.0019752	Carboxylic acid metabolic process	83	4.80E‐39
GO.0009056	Catabolic process	118	1.20E‐38
GO.0071704	Organic substance metabolic process	266	6.39E‐38
GO.1901135	Carbohydrate derivative metabolic process	86	5.19E‐34
GO.0044237	Cellular metabolic process	254	5.57E‐34
GO.0008610	Lipid biosynthetic process	61	6.05E‐31
GO.1901137	Carbohydrate derivative biosynthetic process	66	6.58E‐31
GO.0044283	Small molecule biosynthetic process	54	2.47E‐30
GO.0044248	Cellular catabolic process	95	2.60E‐29
GO.0008152	Metabolic process	263	1.35E‐28
GO.0009058	Biosynthetic process	178	4.28E‐28
GO.0005975	Carbohydrate metabolic process	69	2.76E‐27
GO.0044249	Cellular biosynthetic process	173	2.76E‐27
GO.0032787	Monocarboxylic acid metabolic process	55	4.56E‐27
GO.1901576	Organic substance biosynthetic process	173	1.82E‐26
GO.0044712	Single‐organism catabolic process	71	5.20E‐26
GO.0044763	Single‐organism cellular process	265	1.27E‐25
GO.1901615	Organic hydroxy compound metabolic process	52	1.20E‐23
GO.0055114	Oxidation–reduction process	72	1.21E‐23
GO.0019637	Organophosphate metabolic process	63	2.10E‐22
GO.0090407	Organophosphate biosynthetic process	49	2.10E‐22
GO.0006955	Immune response	79	3.06E‐21
GO.0002376	Immune system process	98	5.71E‐21
GO.0046394	Carboxylic acid biosynthetic process	37	7.43E‐21
GO.0006066	Alcohol metabolic process	42	9.87E‐21
GO.0009260	Ribonucleotide biosynthetic process	29	1.32E‐20
GO.0019538	Protein metabolic process	145	1.36E‐20
GO.0006164	Purine nucleotide biosynthetic process	28	3.18E‐20
GO.0009152	Purine ribonucleotide biosynthetic process	27	1.61E‐19
GO.0045087	Innate immune response	64	1.69E‐19
GO.0006807	Nitrogen compound metabolic process	174	4.53E‐19

*Notes*

The table is generated by inputting the complete protein list from the duck peptide array into the GO database to generate a representative list of GO Biological Processes. Thus, these Biological Processes are represented on the peptide array.

^a^GO ID Gene Ontology database identification number. ^b^The number of proteins refers to the total number of proteins from the complete array list involved in the referenced biological process. ^c^Pathway description designates the biological process identified as relevant. Top 42 shown. ^d^False discovery rate is the likelihood that the biological process named was identified incorrectly.

## 4 DISCUSSION

Although we found limited homology between the mallard 15mer phosphosites and those of black duck in our blastp search (Table 4), we believe this is predominantly due to the limited nature of the data available for black duck. When data were available in the black duck for genes and proteins, the sequences were very nearly identical between the two species (Tables 1 and 2). These results are consistent with the numerous studies highlighting the significant similarities between the black duck and mallard (Avise, Ankney, & Nelson, 1990; Mank et al., 2004).

Although there is a limited amount of sequence data available for the black duck, the very high amount of identity between black duck and mallard suggested that phosphosites found in mallard are also likely to be found in black duck based on our protein–protein and gene–gene comparisons. Therefore, we have developed what we consider to be a “cross‐reactive” array with sites that should produce a signal in both species.

Significant research has been published applying the species‐specific kinome peptide array to a variety of agriculturally relevant species. These studies have spanned research areas including infectious disease (Kogut, Swaggerty, Byrd, Selvaraj, & Arsenault, 2016), gut health (Arsenault & Kogut, 2015), muscle metabolism (Arsenault, Napper, & Kogut, 2013), environmental stress (Napper et al., 2015), handling stress (Chen et al., 2016), and nutrition (Arsenault, Lee, Latham, Carter, & Kogut, 2017). Here, we report the first design of a species‐specific peptide array for a wild species. Much like in agricultural animal researchers, wild animal researchers have a paucity of high‐throughput tools available at their disposal. Most new research tools and reagents are designed for human or mouse application. This duck‐specific kinome peptide array can provide valuable insight into biology in a variety of experimental contexts.

Concern regarding the conservation of the American black duck, especially in the Northeast United States, has led to significant research on their relative decline. While areas further north in Canada have seen the black duck survive and even thrive further south their numbers have dropped, especially when compared to their close relative the mallard (Maisonneuve et al., 2006). In addition, both species of wild duck are known vectors for the spread of AI. A new high‐throughput research tool for the study of both species may provide valuable insight into the biology of both species and provide a ready means of comparison of biological response that may explain some of the differences in species’ responses that have been observed.

## 5 CONCLUSIONS

We have designed and created a new tool for studying waterfowl immunometabolism responses to pathogens, contaminants, and environmental stressors. The tool is species‐specific for *Anas* ducks and yields a molecular level analysis using kinome profiling. A genetic and proteomic comparison of mallard duck and American black duck showed significant similarity between the two species. This tool can be used to compare the responses of mallard duck and American black duck to key environmental variables like contaminants, diet change, and disturbance associated with human activity, which are speculated to underlie the black duck's severe population decline in the mid‐Atlantic region. This tool can also be used to investigate response to pathogens, and we have work in progress to use it to investigate duck immune response to infection with highly pathogenic avian influenza.

## CONFLICT OF INTEREST

None declared.

## AUTHOR CONTRIBUTION

RJA and DCH conceived the ideas and led the writing of the manuscript. RJA designed the methodology and led the creation of the tools. GP and CNJ collected the data. The authors declare that we have no conflict of interest. All authors gave final approval for publication.

## DATA ACCESSIBILITY

The mallard and black duck genomic data used in this study were published in 2013 and deposited at the NIH GenBank. Genome sequence of the duck (*Anas platyrhynchos*). GigaScience Database. https://doi.org/10.5524/101001. All data produced in the course of this work and referred to in the manuscript are published in the manuscript itself or as supplementary data.
